# The greening of Northwest Indian subcontinent and reduction of dust abundance resulting from Indian summer monsoon revival

**DOI:** 10.1038/s41598-018-23055-5

**Published:** 2018-03-15

**Authors:** Qinjian Jin, Chien Wang

**Affiliations:** Center for Global Change Science, Massachusetts Institute of Technology, Cambridge, Massachusetts, 02139 USA

## Abstract

The trends of both rainfall and circulation strength of the Indian summer monsoon has been reviving since 2002. Here, using observational data, we demonstrate a statistically significant greening over the Northwest Indian Subcontinent and a consequent decline in dust abundance due to the monsoon revival. The enhanced monsoonal rainfall causes an increase in soil moisture, which results in a significant greening in the Northwest Indian Subcontinent. These increases in rainfall, soil moisture, and vegetation together lead to a substantial reduction of the dust abundance in this region, especially the Thar Desert, as shown by a negative trend in satellite-retrieved aerosol optical depth. The monsoonal rainfall-induced trends in vegetation growth and dust abundance in the Northwest Indian Subcontinent have important implications for agriculture production and air quality given the projected increases and a westward expansion of the global summer monsoon rainfall at the end of this century.

## Introduction

Climate change might exert more substantial impacts on ecosystem in the arid and semi-arid regions than in the humid regions, owing to the high sensitivities and vulnerabilities to rainfall variations of the former^1–4^. The summer monsoon system in the Northern Hemisphere as a whole has been intensified since the late 1980s^5^. One of its subsystem, the Indian summer monsoon (ISM) has also been experiencing a late while significant revival in both rainfall and circulation during the past 16 years^6^. The ISM revival brings more rainfall to the central, northern, and northwestern parts of the Indian Subcontinent, resulting in a northwestward advance of the monsoonal rainfall distribution^6^, which could cause more floods in the humid regions while potential greening of land surface (i.e., increasing vegetation greenness) in the arid and semi-arid regions.

The Northwest Indian Subcontinent (NWIS), here referred to a region including Southeast Afghanistan, Pakistan, and northwestern India, covers a large area of arid and semi-arid regions as well as several deserts, such as the Thar Desert and the Kharan Desert (Figure S1). Due to extremely dry climate and strong winds, the NWIS suffers heavy and frequent dust storms in spring and summer^7,8^. These dust storms can travel long-distance to North India^9,10^ and the Arabian Sea^11^, degrading air quality and modifying ocean biogeochemistry^12^. Dust aerosols can absorb solar radiation and thus influence local radiation budget^13^, circulations and the ISM rainfall through aerosol direct effect^11,14^.

As the ISM revival expands rainfall distribution further northwestward, the NWIS could receive more rainfall and experience consequent changes in vegetation growth and dust abundance. Here, using various *in situ*, satellite, and reanalysis data, we estimate firstly the changes of the ISM rainfall since 2000 in the NWIS, and then rainfall-induced changes in soil moisture and vegetation growth. Lastly, the changes of aerosol optical depth (AOD) in the NWIS, which are mainly attributed to changes in dust concentrations, are analyzed.

## Results

### The ISM Rainfall Revival

**Figure 1:**
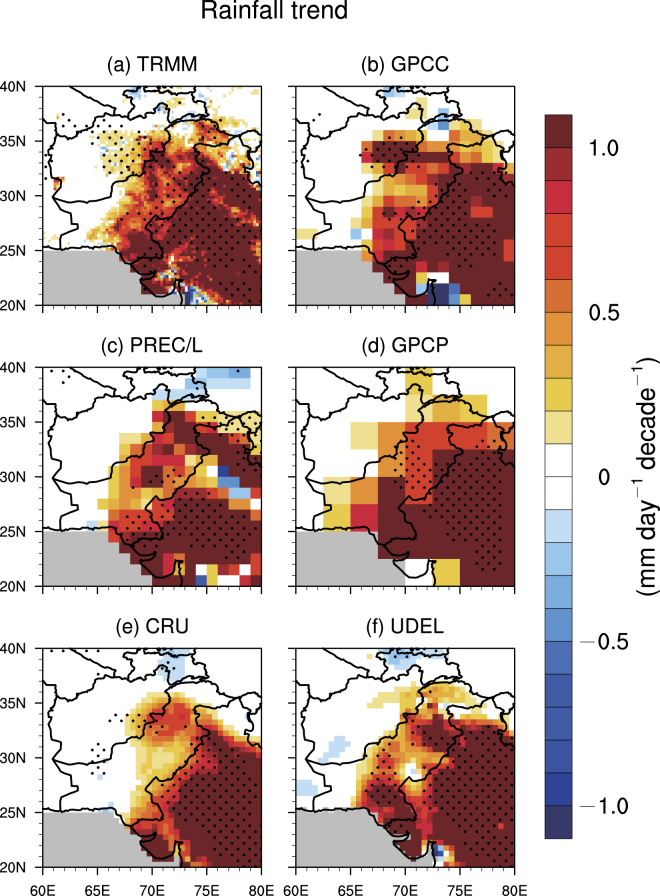
Rainfall trends. The linear trends of the Indian summer monsoon rainfall (mm day^−1^ decade^−1^) in Northwestern Indian subcontinent from 2000 to 2015 for (**a**) TRMM, (**b**) GPCC, (**c**) PREC/L, (**d**) GPCP, (**e**) CRU, and (**f**) UDEL. The black dots represent grid points that are statistically significant over the 95% confidence level. Grey colors indicate missing values. The figure was created using the National Center for Atmospheric Research Command Language (NCL) (version 6.4.0) [Software]. (2017). Boulder, Colorado: UCAR/NCAR/CISL/TDD. http://dx.doi.org/10.5065/D6WD3XH5.

Though the ISM rainfall revival covers a large area over the subcontinent, we focus on the NWIS characterized by semi-arid and arid climate, where vegetation growth is very sensitive to rainfall variations while mineral dust emissions are active and the dust particles can be transported eastward to the central and north Indian. Figure 1 demonstrates the linear trends of the ISM rainfall (defined as June–July–August–September) during the past 16 years using 6 data sets (see Methods). Specifically, TRMM data show statistically significant positive trends of rainfall in the NWIS at 95% confidence level. The strongest positive trends are observed in northwestern India with a magnitude greater than 1 mm day^−1^ decade^−1^. Note that in most parts of the Thar Desert, rainfall has positive trends with magnitudes larger than 1 mm day^−1^ decade^−1^, which is very unusual and has great implications for mineral dust abundance (to be discussed later). The positive rainfall trends are also observed in the entire Pakistan except the southwestern part, but the magnitudes—0.5 to 0.8 mm day^−1^ decade^−1^—are weaker than those in northwestern India. In a small area of eastern Afghanistan, much weaker positive trends are seen. The other five datasets also illustrate very similar trend patterns to those in the TRMM data, but have fewer grid points passing significant test at the 95% confidence level particularly in Pakistan and Afghanistan, likely owing to their relatively sparsely-distributed base observational stations over the analyzed region. There is a large southeast–northwest gradient in the positive rainfall trends, which is consistent with the monsoon seasonal progression. The positive rainfall trends in the NWIS clearly indicates a northwestward expansion of the monsoon system.

**Figure 2:**
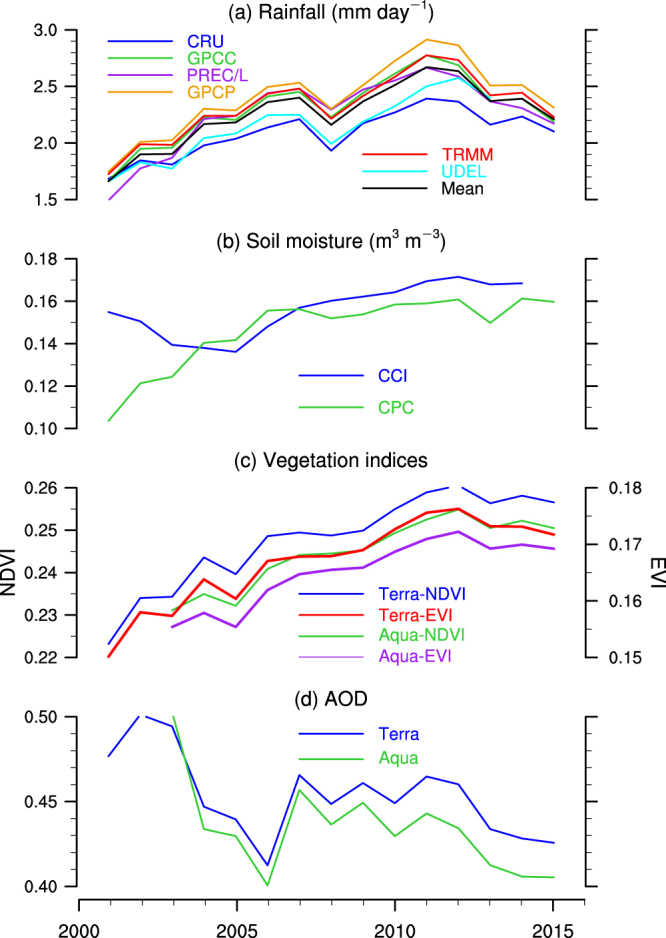
Time series. Three-year running averaged time series for (**a**) rainfall (mm day^−1^), (**b**) soil moisture (m^3^ m^−3^), (**c**) vegetation indices, and (**d**) AOD during monsoon season from 2001/2003 to 2013/2015. All time series are area-averaged in the entire domain (20°N–40°N, 60°E–80°E). The figure was created using the National Center for Atmospheric Research Command Language (NCL) (version 6.4.0) [Software]. (2017). Boulder, Colorado: UCAR/NCAR/CISL/TDD. http://dx.doi.org/10.5065/D6WD3XH5.

The rainfall in the NWIS as a whole has increased by 27% since 2000 based on the TRMM data. Other data sets show an increase from 31% to 38%, with an ensemble mean of 30% (Figure S2). Note that the rainfall in the NWIS shows a lightly negative trend starting in 2013 (Fig. 2), reflecting the impact of the 2014 drought in India that is partially caused by the teleconnection between sea surface temperatures (SSTs) over the northern Indian Ocean and the tropical western Pacific through the Indonesian Throughflow in the upper ocean^15^.

### Soil Wetting in the NWIS

**Figure 3:**
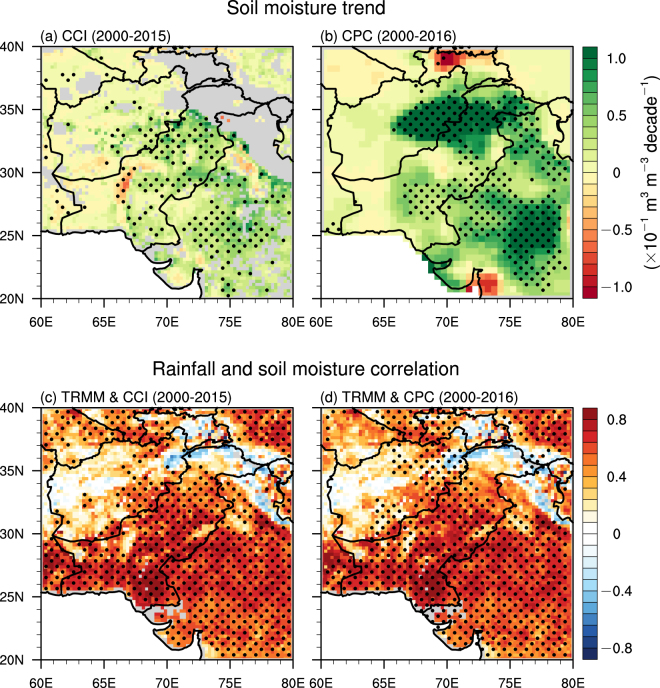
Soil moisture trend and rainfall–soil moisture correlation. Top row: the linear trends of soil moisture (×10^−1^ m^3^ m^−3^ decade^−1^) during the ISM season from 2000 to 2015/2016 for (**a**) CCI and (**b**) CPC. Bottom row: the Pearson linear correlation between rainfall and soil moisture from 2000 to 2015/2016 for (**c**) TRMM and CCI and (**d**) TRMM and CPC. The black dots represent grid points that are statistically significant over the 95% confidence level. Grey and white colors indicate missing values. The figure was created using the National Center for Atmospheric Research Command Language (NCL) (version 6.4.0) [Software]. (2017). Boulder, Colorado: UCAR/NCAR/CISL/TDD. http://dx.doi.org/10.5065/D6WD3XH5.

Monsoon rainfall revival could increase soil moisture. Figure 3 illustrates the linear trends of soil moisture during monsoon season from 2000 to 2015/2016. The retrieved surface soil moisture (i.e., between 0.5 to 2.0 cm) from satellite remote sensing data displays significant positive trends at 95% confidence level in northwestern India, central Pakistan, and eastern Afghanistan with a magnitude around 0.5 × 10^−1^ m^3^ m^−3^ decade^−1^ (Fig. 3a). There are many missing values in the remote sensing data due to the retrieval algorithm. In order to cross-evaluate the robustness of the observed positive trends in soil moisture, another independent model dataset is also used in our analysis (Fig. 3b; see also Methods). The modelled soil moisture has a larger spatial coverage including the areas with missing remote-sensing data. The spatial patterns in the model data are very similar to those in remote-sensing data but with larger magnitudes almost in the entire domain. The model data also display strong positive trends of soil moisture in the northern parts of Afghanistan and Pakistan and northwestern India with magnitudes close to or larger than 1 × 10^−1^ m^3^ m^−3^ decade^−1^. Overall, the remote sensing data and the model data respectively indicates an increase in soil moisture by 17% and 27% in the entire domain. Note that the spatial patterns of soil moisture trends are generally consistent with these of rainfall, showing a southeast–northwest gradient, indicating the high sensitivity of soil moisture to rainfall in this region. The relationship between rainfall and soil moisture is also reflected by the significant positive correlation between them in the entire domain particularly over regions with substantial positive rainfall trends (see Fig. 1), indicating the clear response of soil moisture to the monsoonal rainfall reversal. The exceptional cases are only seen in the mountainous regions, such as the southwestern slope of the Tibetan Plateau and West and Central Pakistan (Fig. 3).

### Rainfall Impacts on the Greening of the NWIS

Since soil moisture dominates vegetation growth in the arid and semi-arid regions, the rainfall revival that has increased soil moisture thus could further lead to changes in vegetation growth. The impact of rainfall on vegetation growth can be depicted by the correlation between them. Figure S3 demonstrates the linear correlation coefficients between TRMM rainfall and MODIS vegetation indices during the monsoon season from 2000 and 2016. Here, the correlation coefficients are calculated based on the deseasonalized monthly data. Significant correlation coefficients between rainfall and vegetation indices with magnitudes ranging from 0.4 to 0.8 are found in northwestern India, central Pakistan, and a small area of eastern Afghanistan. Again, the spatial patterns of above correlation are very similar to the patterns of the positive trends of rainfall and soil moisture in the entire domain. The only exception is western central India (in the lower-right corner of the domain), mainly due to the abundant rainfall there, which results in a weak dependence of vegetation growth on rainfall in this wet region. The characteristics of the spatial patterns of correlation coefficients between rainfall and vegetation indices support the previous findings that vegetation growth in arid and semi-arid regions is dominated by rainfall^16,17^.

**Figure 4:**
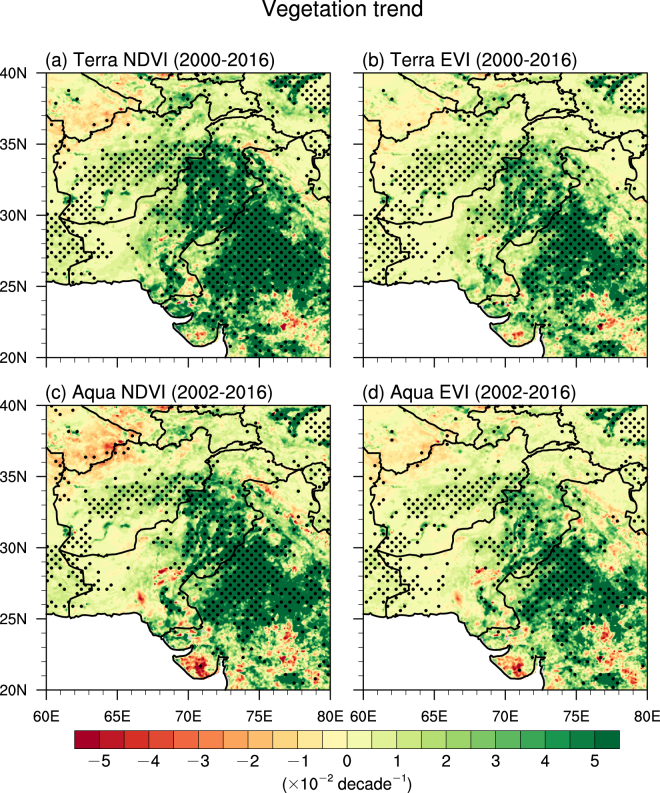
Vegetation trends. The linear trends of vegetation indices (×10^−2^ decade^−1^) during the ISM season from 2001/2003 to 2015 for (**a**) MODIS-Terra NDVI, (**b**) MODIS-Terra EVI, (**c**) MODIS-Aqua NDVI, and (**d**) MODIS-Aqua EVI. The black dots represent grid points that are statistically significant over the 95% confidence level. White colors indicate missing values. The vegetation data were retrieved from the NASA Land Processes Distributed Active Archive Center (LP DAAC), USGS/Earth Resources Observation and Science (EROS) Center, Sioux Falls, South Dakota, https://lpdaac.usgs.gov/data_access/data_pool. The figure was created using the National Center for Atmospheric Research Command Language (NCL) (version 6.4.0) [Software]. (2017). Boulder, Colorado: UCAR/NCAR/CISL/TDD. http://dx.doi.org/10.5065/D6WD3XH5.

Due to the monsoon revival and the associated northwestward expansion of the monsoonal rainfall as well as the strong positive correlation between rainfall and vegetation indices, the NWIS depicts a widespread greening. Figure 4 shows the linear trends of MODIS vegetation indices during monsoon season from 2000 to 2016. Significant positive trends of NDVI are observed in northwestern India that covers a large part of the Thar Desert with magnitudes greater than 5 × 10^−2^ decade^−1^ (Fig. 4a). Relatively weaker positive trends are seen in almost the entire Pakistan with magnitudes from 3 × 10^−2^ to 5 × 10^−2^ decade^−1^. The weakest positive trends are detected in Afghanistan and southeastern Iran with magnitude around 1 × 10^−2^ decade^−1^. Again, the greening also shows a southeast–northwest gradient similar to the trends in rainfall and soil moisture. The spatial patterns of NDVI trends in MODIS-Terra are similar to those in MODIS-Aqua. On the other hand, the magnitudes of NDVI trends are generally greater than EVI trends in both MODIS-Terra and MODIS-Aqua data. Overall, vegetation indices increased by 14% to 21% in the entire domain during our analysis period. Note that the vegetation indices demonstrate a weak decrease after 2012 (Fig. 2), which is consistent with the variations in rainfall and soil moisture.

### Reduction in AOD

**Figure 5:**
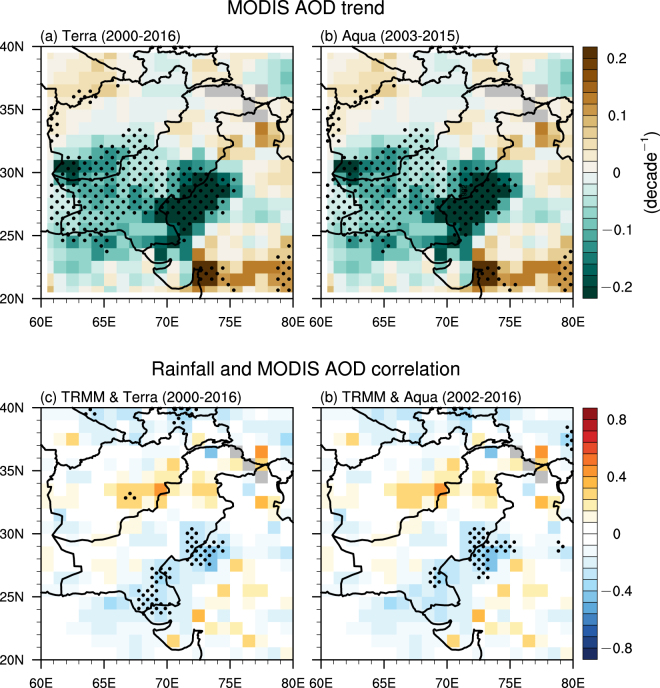
AOD trends and rainfall–AOD correlation. Top row: the linear trends of AOD (decade^−1^) during the ISM season from 2001/2003 to 2015 for (**a**) MODIS-Terra and (**b**) MODIS-Aqua. Bottom row: the Pearson linear correlation between rainfall and AOD from 2000/2002 to 2016 for (**c**) TRAMM and Terra AOD and (**d**) TRMM and Aqua AOD. The black dots represent grid points that are statistically significant over the 95% confidence level. Grey colors indicate missing values. The aerosol data were retrieved from the NASA Level-1 and Atmosphere Archive & Distribution System (LAADS) Distributed Active Archive Center (DAAC) at the Goddard Space Flight Center (GSFC), https://ladsweb.modaps.eosdis.nasa.gov/. The figure was created using the National Center for Atmospheric Research Command Language (NCL) (version 6.4.0) [Software]. (2017). Boulder, Colorado: UCAR/NCAR/CISL/TDD. http://dx.doi.org/10.5065/D6WD3XH5.

All of the above increases in rainfall, soil moisture, and vegetation indices can reduce mineral dust emissions in deserts and dust abundance within the domain of analysis. Several physical mechanisms can facilitate such reductions, including increased bounding forces among soil particles due to wet soil that could reduce dust emissions, and increases in wet scavenging of dust by enhanced precipitation and increases in interception of suspended soil particles by more densely vegetation that both could directly reduce dust abundance in the atmosphere. Figure 5 shows the linear trends of AOD in summer monsoon season from 2000/2002 to 2016. The strongest negative trends are seen in the Thar Desert with magnitudes greater than 0.2 decade^−1^ where the trends of rainfall, soil moisture, and vegetation indices are high but not the highest, supporting the hypotheses that the negative AOD trends are caused mainly by reduction in dust emissions that are dominated by changes in rainfall, soil moisture, and vegetation. Relatively weaker decreasing trends are detected in southern Pakistan and southeastern Afghanistan with magnitudes around 0.1 decade^−1^. Significant negative trends are also observed over a small area in northeastern Arabian Sea due to transport of dust aerosols from dust source regions to the Arabian Sea. Sparsely-distributed positive trends are detected in the lower-right and upper-left corners of the domain (Fig. 5), which are consistent with the negative trends of vegetation greenness near these regions. AOD shows a strong decrease before 2006 already, such a decrease has been continued though in a slower rate afterwards (Fig. 2). Over the entire domain, AOD has decreased by 11% and 22% according to MODIS-Terra and MODIS-Aqua, respectively. The area-averaged trends of all variables in the entire domain are summarized in Table S1 for changes in absolute value and Figure S2 for changes in percentage. Note that wet scavenging of aerosols appears to be not a main factor contributing to the negative AOD trends, because negative AOD trends would otherwise be detected in more regions, particularly in the lower right corner of the domain where the strongest positive rainfall trends are seen.

The proposed physical mechanism behind the rainfall-induced reduction in AOD is also supported by the significantly negative linear correlation between rainfall and AOD in dust source region, i.e., the Thar Desert (Fig. 5). Note that this negative correlation between rainfall and AOD is only detected in dust source region rather than in the entire domain, suggesting that rainfall reduces aerosol abundance mainly through prohibiting dust emissions instead of wet scavenging. Further analysis shows no significant trends of wind speed in the entire domain during the past 15 years (Figure S4), implying that rainfall and the associated changes in soil moisture and vegetation rather than wind have dominated the reduction of AOD.

## Conclusions and Discussion

Using multiple datasets from *in situ* observations and remote sensing, we find that the ISM rainfall has been increased by about 30% in the NWIS since the monsoon revival around 2000, and the rainfall distribution has clearly had a northwestward expansion. A high sensitivity of vegetation growth to the variations of the monsoonal rainfall in the desert, arid, and semi-arid regions is reflected from their close correlation (with a coefficient of 0.6). Therefore, due to the increases in the monsoonal rainfall and the consequent wetting of soil, a significant widespread greening is found in the NWIS with a magnitude from 14% to 21%, particularly in the Thar Desert and other deserts in the NWIS. It is worth mentioning that the spatial patterns of the positive trends in rainfall, soil moisture, and vegetation show a southeast–northwest gradient, possibly owing to the seasonal evolution of the monsoonal intensity. In addition to changes in vegetation greenness induced by monsoon revival, dust abundance reflected in AOD also demonstrate significant decreases in the NWIS by 11% to 22%, especially over the desert regions. Further analysis shows no significant trend of wind speed in the entire domain. Based on the above findings, the main physical mechanism behind the decreasing trend of AOD is the ISM revival-induced increase in soil moisture, which in turn benefits vegetation growth and further prevents dust emissions in the source regions. It should also be pointed out that a reasonable estimation of the contribution of wet scavenging to AOD decreasing trends can be hardly obtained without model simulations. However, current climate models have limited capability to capture the observed inter-annual variabilities and decadal trends of dust emissions^18–20^. Therefore, this study does not directly quantify the impact of wet scavenging on AOD trends. The result of this analysis only implies that wet scavenging does not play a dominant role in causing negative AOD trends.

The rainfall trend revival of the ISM from a half-century drying to a persistent wetting since 2002 is an important phenomenon, and must have already impacted on many aspects of regional and global climate, ranging from changes in land surface to dust abundance as demonstrated in this study, and furthermore to impact on agriculture production in South Asia^21^, causing more extreme rainfall events^22,23^, transporting more water to the lower stratosphere through the strengthened monsoonal deep convections^24^, increasing water vapor advection over the Tibetan Plateau and causing greening of the plateau^25–27^, and generating more catastrophic tropical cyclones^28^, and so forth. All of these potential impacts induced by the ISM revival should be addressed in future studies. Looking into future, climate models participating the Coupled Model Intercomparison Project Phase 5 project a significant enhanced rainfall and a westward expansion of global summer monsoon over land at the end of the 21^st^ century^29,30^, which, according to this study, would have strong implications of greening and reduction in dust aerosols over arid and semi-arid regions in North African, Middle East, and Central Asia. Finally, the gap between the projections of the enhanced and westward expansion of the global monsoonal rainfall^29,30^ and the accelerated dryland expansion^2^ should also be addressed in future studies.

## Methods

This section describes all the data sets and the statistical methods used in this study. A summary of these data sets can be found in Table S1.

### Rainfall

In order to evaluate the uncertainties induced by data, six rainfall datasets are used, including rainfall data from the Climate Research Unit (CRU) with version 4.01 (0.5° × 0.5°)^31^, the Global Precipitation Climatology Centre with version 7 (GPCC; 1° × 1°)^32^, United States National Oceanic and Atmospheric Administration (PREC/L; 1° × 1°)^33^, the Global Precipitation Climatology Project (GPCP) with version 02 (2.5° × 2.5°)^34^, the Tropical Rainfall Measuring Mission (TRMM) with version 3B43 (0.25° × 0.25°)^35^, and the University of Delaware with version 4.01 (UDEL; 0.5° × 0.5°)^36^. In addition, the arithmetic mean of the six data sets is calculated as the ensemble mean.

### Soil Moisture

Two data sets of soil moisture are used. The first data set is from the European Space Agency Climate Change Initiative (CCI) project that provides long-term consistent time series of soil moisture covering the globe. The CCI soil moisture data are retrieved based on satellite-borne passive and active microwave sensors with a spatial resolution of 0.25° × 0.25° in the depth between 0.5 to 2.0 cm^37^. The current CCI soil moisture data of version 032 in phase 1 does not yet incorporate microwave sensors of the Soil Moisture and Ocean Salinity mission and the Soil Moisture Active and Passive mission, which will be included in the next phase(s) of CCI products. Another soil moisture data set used in this study is modeled results produced using the National Weather Service Global Forecast System (GFS) model at the Climate Prediction Center (CPC) of the National Oceanic and Atmospheric Administration^38^. The CPC soil moisture of version 2 is used with a spatial resolution of 0.5° × 0.5° in a depth to 1.6 m.

### Vegetation

The Moderate Resolution Imaging Spectroradiometer (MODIS) aboard on Terra and Aqua satellites observes Earth’s atmosphere, land, and ocean at 36 wavelengths from 0.405 μm to 14.385 μm, with a wide swath of 2,330 km, enabling detection of many characteristics of Earth with a global coverage time of one to two days. Two MODIS land products have been retrieved—the Normalized Difference Vegetation Index (NDVI) and the Enhanced Vegetation Index (EVI). Comparing to NDVI, EVI minimizes the canopy–soil variations and improves sensitivity over densely vegetated areas^39^. Here, we use monthly NDVI and EVI data in collection 6 from MODIS onboard both Terra (MOD13C2) and Aqua (MYD13C2) satellites, with a spatial resolution of 0.05° × 0.05°.

### Aerosol

AOD is widely used to represent the abundance of aerosols integrated in the entire atmospheric column. Both MODIS-Terra and MODIS-Aqua provide two AOD products at 550 nm—the “dark target” and the “deep blue”, differing in the ways of how to account for the land surface reflectance^40^. The former uses 0.47 μm, 0.67 μm, 2.1 μm channels to estimate surface reflectance and thus works best over dark vegetated and ocean surfaces but bright surfaces; while the latter uses the blue channels, and works best over bright surfaces (e.g., deserts) but can also work on dark surfaces. Here, the combined monthly AOD of the “dark target” and the “deep blue” of collection 6 are employed with a spatial resolution of 1° × 1° from both Terra (MOD08_M3) and Aqua (MYD08_M3). The newly released collection 6 of AOD products applied significant changes in the retrieval algorithm comparing to collection 5, including but not limited to an improved cloud screening scheme and a dynamic surface reflectance database to replace the previous static lookup tables^41^. Consequently, the spatial coverage and accuracy of AOD in collection 6 have been significantly improved^42^.

### Wind

The wind speed at 10 m above the surface are from the Modern Era-Retrospective Analysis for Research and Applications^43^ (MERRA; 1/2° latitude × 2/3° longitude) and the European Centre for Medium-Range Weather Forecasts (ECMWF) Interim Reanalysis^44^ (ERAI; 3/4° latitude × 3/4° longitude) global reanalysis. Both data sets span from 2000 to 2015.

### Statistical Analysis

The linear trends are estimated using the Theil-Sen method based on 3-year running averages to reduce the impact of the large inter-annual variabilities on the trend analysis. The Mann-Kendall test, which is a non-parametric (i.e., distribution free) test, are employed to calculate the confidence level of the linear trends^45^. The Pearson linear correlation coefficients are calculated and the associated statistical significance is determined using the two-sided *r*-test.

## Electronic supplementary material

Supplementary Information
