# Supplementary material for: The greening of Northwest Indian subcontinent and reduction of dust abundance resulting from Indian summer monsoon revival
Source: Sci Rep. 2018 Mar 15;8:4573. doi: 10.1038/s41598-018-23055-5 (PMC5854704; doi:10.1038/s41598-018-23055-5)
Supplement: Supplementary file 1 — Supplementary Information [file 41598_2018_23055_MOESM1_ESM.docx]

Supplementary Information for

**The greening of Northwest Indian subcontinent and reduction of dust abundance resulting from Indian summer monsoon revival**

Qinjian Jin^1^ and Chien Wang^1^

^1^Center for Global Change Science, Massachusetts Institute of Technology, Cambridge, Massachusetts 02139, USA

*Correspondence to*: Qinjian Jin (jqj@mit.edu)

**
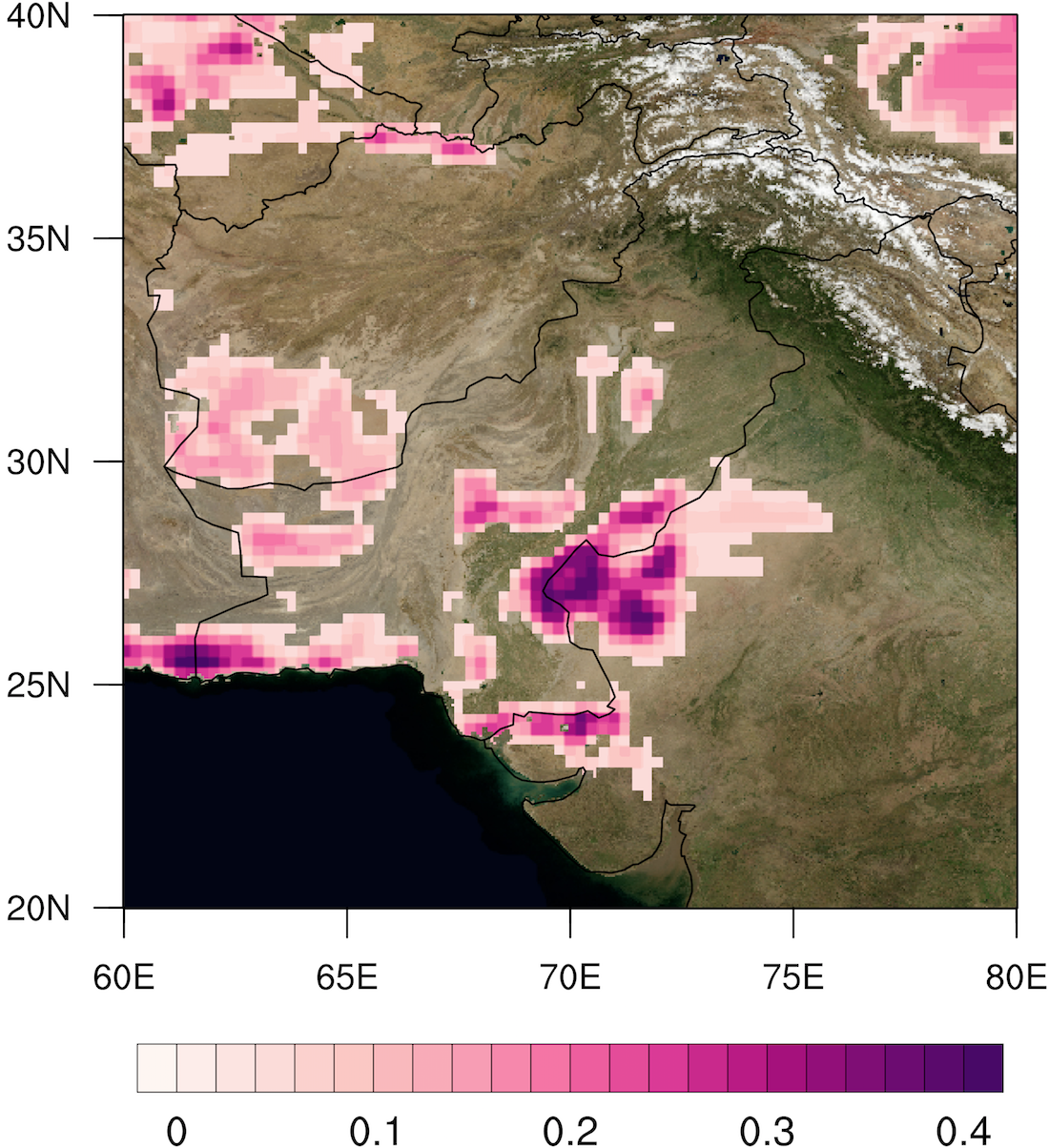
**

**Figure S1.** Study domain. Contours represent the spatial patterns of erodibility that is a measure of the susceptibility of soil particles to detachment from the surface and ejection into the air by strong winds. Data are retrieved based on topography^1^. The base map is a true color Earth image from NASA. The base map is from NASA Visible Earth (https://visibleearth.nasa.gov/view.php?id=74092). The figure was created using the National Center for Atmospheric Research Command Language (NCL) (version 6.4.0) [Software]. (2017). Boulder, Colorado: UCAR/NCAR/CISL/TDD. http://dx.doi.org/10.5065/D6WD3XH5.

**Figure S2.** Changes in percent. The changes in percent area-averaged over the study domain during 2001/2003 to 2013/2015 for rainfall, soil moisture, vegetation, and AOD. The figure was created using the National Center for Atmospheric Research Command Language (NCL) (version 6.4.0) [Software]. (2017). Boulder, Colorado: UCAR/NCAR/CISL/TDD. http://dx.doi.org/10.5065/D6WD3XH5.

**Figure S3.** Spatial patterns of linear correlation coefficients between rainfall and vegetation indices during the Indian summer monsoon season from 2000/2002 to 2016. The correlation coefficients are calculated based on deseasonalized monthly data. The black dots represent the grid points that are statistically significant above the 95% confidence level. Grey colors indicate missing values. The figure was created using the National Center for Atmospheric Research Command Language (NCL) (version 6.4.0) [Software]. (2017). Boulder, Colorado: UCAR/NCAR/CISL/TDD. http://dx.doi.org/10.5065/D6WD3XH5.

**Figure S4.** Wind speed trends. The linear trends of wind speed (m s^−1^ decade^−1^) during the ISM season from 2001/2003 to 2015. The data are from the Modern Era-Retrospective Analysis for Research and Applications^2^ (MERRA; 1/2° latitude × 2/3° longitude) and the European Centre for Medium-Range Weather Forecasts (ECMWF) Interim Reanalysis^3^ (ERAI; 3/4° latitude × 3/4° longitude) global reanalysis. The green dots represent grid points that are statistically significant over the 95% confidence level. The figure was created using the National Center for Atmospheric Research Command Language (NCL) (version 6.4.0) [Software]. (2017). Boulder, Colorado: UCAR/NCAR/CISL/TDD. http://dx.doi.org/10.5065/D6WD3XH5.

**Table S1. Summary of data sets and the corresponding trends**. One star (“*”), two stars (“**”), and three stars (“***”) respectively represent trends that are significant at 90%, 95%, and 99% confidence level. “N.A.” means “not applicable”.

| Variables | Data | Trends | Resolution | Duration |
| --- | --- | --- | --- | --- |
| Rainfall  (mm day^−1^ decade^−1^) | CRU | 0.41** | 0.5°×0.5° | 2000–2016 |
|  | GPCC | 0.49** | 1°×1° | 2000–2016 |
|  | PREC/L | 0.48** | 1°×1° | 2000–2016 |
|  | GPCP | 0.58** | 2.5°×2.5° | 2000–2016 |
|  | TRMM | 0.46** | 0.25°×0.25° | 2000–2016 |
|  | UDEL | 0.47 | 0.5°×0.5° | 2000–2014 |
|  | Mean | 0.47** | N.A. | 2000–2016 |
| Soil moisture  (m^3^ m^−3^ decade^−1^) | CCI | 0.017* | 0.25°×0.25° | 2000-2015 |
|  | CPC | 0.027*** | 0.5°×0.5° | 2000-2016 |
| Vegetation index  (decade^−1^) | Terra-NDVI | 0.021*** | 0.05°×0.05° | 2000-2016 |
|  | Terra-EVI | 0.015*** | 0.05°×0.05° | 2000-2016 |
|  | Aqua-NDVI | 0.024*** | 0.05°×0.05° | 2002-2016 |
|  | Aqua-EVI | 0.018*** | 0.05°×0.05° | 2002-2016 |
| AOD  (decade^−1^) | Terra | −0.04** | 1°×1° | 2000-2016 |
|  | Aqua | −0.07** | 1°×1° | 2002-2016 |

**References**

1. Ginoux P*, et al.* Sources and distributions of dust aerosols simulated with the GOCART model. *J Geophys Res-Atmos* **106**, 20255-20273 (2001).

2. Rienecker MM*, et al.* MERRA: NASA's Modern-Era Retrospective Analysis for Research and Applications. *Journal of Climate* **24**, 3624-3648 (2011).

3. Dee DP*, et al.* The ERA-Interim reanalysis: configuration and performance of the data assimilation system. *Q J Roy Meteor Soc* **137**, 553-597 (2011).
